# A Novel SNP in the Promoter Region of *IGF1* Associated With Yunshang Black Goat Kidding Number *via* Promoting Transcription Activity by SP1

**DOI:** 10.3389/fcell.2022.873095

**Published:** 2022-05-12

**Authors:** Kunyu Li, Yufang Liu, Xiaoyun He, Lin Tao, Yanting Jiang, Rong Lan, Qionghua Hong, Mingxing Chu

**Affiliations:** ^1^ Key Laboratory of Animal Genetics, Breeding and Reproduction of Ministry of Agriculture and Rural Affairs, Institute of Animal Science, Chinese Academy of Agricultural Sciences, Beijing, China; ^2^ College of Life Science and Food Engineering, Hebei University of Engineering, Handan, China; ^3^ Yunnan Animal Science and Veterinary Institute, Kunming, China

**Keywords:** Goat, kidding number, granulosa cell proliferation, IGF1, Sp1, single-nucleotide polymorphism (SNP)

## Abstract

*IGF1*, a member of the insulin-like growth factor (IGF) superfamily, is also known as the growth-promoting factor (somatomedin C). *IGF1* is involved in vertebrate growth and development, immunity, cell metabolism, reproduction, and breeding. However, there are relatively few studies on the relationship between *IGF1* and goat reproduction. In this study, a new transcription factor *SP1* bound to the *IGF1* g. 64943050T>C promoted granulosa cell (GC) proliferation. A mutation g.64943050T>C located in the promoter region of *IGF1* was identified. Association analysis revealed that the kidding number in the first and second litters and the average number of first three litters of the CC genotype (2.206 ± 0.044, 2.254 ± 0.056, and 2.251 ± 0.031) were significantly higher than those in the TC genotype (1.832 ± 0.049, 1.982 ± 0.06, and 1.921 ± 0.034) and TT genotype (1.860 ± 0.090, 1.968 ± 0.117, and 1.924 ± 0.062) (*p* < 0.05). The kidding number in the third litter of the CC genotype (2.355 ± 0.057) was significantly higher than that in the TT genotype (2.000 ± 0.107) (*p* < 0.05). Then, the function of this mutation was validated by the dual-luciferase reporter assay and EMSA. The results showed that the luciferase activity of IGF1-mutant-C was significantly higher than that of IGF1-Wild-T (*p* < 0.05). The EMSA also showed that the binding ability of IGF1-mutant-C was higher than that of IGF1-Wild-T (*p* < 0.05). Subsequently, the transcription factor *SP1* was predicted to bind to the mutation of *IGF1* (g.64943050T>C). Overexpression of SP1 promotes the expression of *IGF1* in the primary granulosa cells (GCs). The results of the CCK-8 assay and the expression of GC proliferation factors (*CDK4*, *cyclin D1*, and *cyclin D2*) demonstrated that *SP1* promoted GC proliferation by regulating *IGF1* expression. Our results suggested that the *IGF1* g.64943050T>C was significantly associated with the kidding number of Yunshang black goats, and *SP1* as a transcription factor of *IGF1* binding to the mutation T>C regulated the expression of *IGF1*. Furthermore, *SP1* promoted goat GC proliferation by regulating the expression of *IGF1*, which provides a new insight for the goat fertility trait.

## Introduction

The carcasses of goats are very lean compared to those of sheep at the same age (Stanišić et al., 2012). Nutritionally, goat meat is an important source of high-quality protein, healthy fats, low calorie intramuscular fats, saturated fats, and sodium. In addition, goat meat has high levels of iron, potassium, and essential amino acids (Mcmillin and Brock, 2005; Givens et al., 2006; Lee et al., 2008; Horcada et al., 2012). However, goats are more difficult to rear than sheep, and their growth rate is generally slower and smaller than that of sheep, making goat meat scarce and significantly more expensive than sheep (Brady et al., 2020). Increasing the kidding number of goats is the easiest and most direct way to increase goat meat production. Compared to traditional breeding methods, molecular genetic breeding has several advantages, including high accuracy, operability, cost saving, and the ability to quickly and efficiently improve goat prolificacy traits.

Insulin-like growth factor 1 (*IGF1*), known as growth-promoting factor (somatomedin C), has insulin-like functions and significant diversity in biological effects because of its molecular structure being extremely similar to insulin (Ma et al., 2015). *IGF1* is conserved across species, and *IGF1* can be selectively spliced into multiple transcripts encoding different precursor IGF1 proteins. In goats, *IGF1* is located on chromosome 5 and contains six exons (Górecki et al., 2007; Xiao et al., 2009). *IGF1* is an important component of the insulin-like growth factor (IGF) superfamily and is commonly expressed in mammalian gonadal axis tissues such as the hypothalamus, anterior pituitary, ovary, oviduct, and uterus (Bach and Bondy, 1992; Mikawa et al., 1995; Adam et al., 2000; Hunter et al., 2004; Filby and Tyler, 2007). IGF1 is mainly secreted by the liver, and GH (growth hormone) secreted by the pituitary acts on the GHR (growth hormone receptor) of the liver to regulate the release of IGF1. IGF1 acts as a systemic growth factor and also acts on the hypothalamus as a negative feedback regulator of growth hormone secretion. Meanwhile, both growth hormone and insulin-like growth factor are affected by binding proteins (GHBP and IGFBP, respectively) (Hunter et al., 2004). IGF1 acts on IGF1 receptors and is generally involved in vertebrate growth and development, organismal immunity, cellular metabolism, and reproduction (Cannata et al., 2010; Ni et al., 2013; Hu and Liu, 2014; Kindler et al., 2016). The receptors of *IGF1* are found in almost every organ of the mammalian reproductive system, regulating reproductive system processes from the center to the organs. *IGF1*, as a hormonal regulator, acts directly on GnRH neurons, stimulating their transcriptional translation. It also acts synergistically with GnRH to promote follicular growth and steroid hormone synthesis, thereby controlling the initiation of the first estrus and reproductive activity in adult mammals (Lucy, 2000; Miller and Gore, 2001). Previous studies have also shown that polymorphisms in *IGF1* are significantly associated with lambing numbers in sheep and goats (He et al., 2012; Thomas et al., 2016; Naicy et al., 2017; Tao et al., 2021). This suggests that *IGF1* plays an important role in regulating the reproductive performance of females.

In this study, we detected an SNP in the promoter region of the *IGF1*, determined the relationship between mutations in the promoter region of the *IGF1* and kidding number of goats, predicted the transcription factor *SP1* bound to this mutation locus, and explored the potential molecular mechanisms between the expression of transcription factors *SP1* and *IGF1* and reproductive performance in goats. These results are important for elucidating the molecular characteristics of *IGF1*, understanding its function in regulating kidding number of goats, and comprehensively exploring the molecular mechanism of the IGF family in regulating the fertility of goats.

## Materials and Methods

### Animals and Sample Collection

Blood was collected from 400 Yunshang black goats (2–5 years old). All the goats were raised in Honghe Hani and Yi Autonomous Prefectures in Yunnan Province, China in the same environment. Genomic DNA was extracted using the phenol chloroform method. DNA sample concentration was detected by Nano Drop 2000 (Thermo Fisher, USA), and DNA quality was detected by 1.2% agarose gel electrophoresis. Ten high- and low- yielding Yunshang black goats were selected, and after euthanasia, ovarian tissues were extracted, put into liquid nitrogen immediately, and then stored at −80°C. RNA was extracted from the tissue samples using the Animal Tissue RNA Extraction Kit (Tiangen, China), and RNA concentration was detected using Nano Drop 2000, and RNA quality was detected by 1.2% agarose gel electrophoresis.

### Reverse Transcription, Real-Time Quantitative PCR, and Genotyping

The cDNA was synthesized using the TaKaRa Reverse Transcription Kit (TaKaRa, Japan). Reverse transcription system: 1.0 μl reverse transcription mixed primer, 1 μl Oligo dT primer, 1 μl random primer, 4 μl fluorescence quantification buffer, 1 μl total RNA, 12 μl double-distilled water, and operated on ice throughout. Reaction conditions: 37°C for 15 min; 85°C for 5 s. The cDNA product after the completion of reverse transcription was diluted, and PCR was performed with the housekeeping gene *RPL19*, and the quality was qualified and stored at −20°C for the detection of gene mRNA expression.

The total volume of the PCR system was 20 μl: 10 μl of Ex Taq primer II, 0.8 μl of the upstream primer, 0.8 μl of the downstream primer, 2 μl of the cDNA template, and 6.4 ul of double-distilled water. *RPL19* was used as the control gene, and the primer sequences are shown in Table 1.

**TABLE 1:** Primers used in this study.

Primers	Primers sequence(5′-3′)	Tm(°C)	Usage
*IGF1*	F:GAAGCAGGTGAAGATGCCAGT	60	primers of IGF1 for real-time PCR
	R:CTTGTTGAAATAAAAGCCCCTG		
*SP1*	F:GCCTCAGGTGATCATGGAGC	60	primers of SP1 for real-time PCR
	R:CAGAGCCCCTTCCTTCACTATC		
*CDK4*	F:GAGCATCCCAATGTTGTCAGG	60	primers of CDK4 for real-time PCR
	R:ACTGGCGCATCAGATCCTTT		
*Cyclin D1*	F:GCCACAGACGTGAAGTTCATTT	60	primers of cyclin D1 for real-time PCR
	R:CGGGTCACATCTGATCACCTT		
*Cyclin D2*	F:ATGTGGATTGCCTCAAAGCC	60	primers of cyclin D2 for real-time PCR
	R:CAGGTCGATATCCCGAACATC		
*RPL19*	F:ATCGCCAATGCCAACTC	60	house-keeping gene, primers of RPL19 for real-time PCR
	R:CCTTTCGCTTACCTATACC		

The *IGF1* was typed using the KASP method using a 384-well plate with a total volume of 10.14 µl. The reaction system included 2.5 µl DNA template, 2.5 µl 2×Master Mix, 0.07 µl primer mix, 2.57 µl working solution, and 2.5 µl RNase-free water blown and mixed and then performed instantaneously at 3,000 rpm. After PCR, the wells were removed, and the data were scanned.

### Bioinformatics Analysis

The binding sites of transcription factors at the location of the mutant site and the transcription factors bound were predicted using Jaspar (http://jaspar.genereg.net/).

### Plasmid Construction and Extraction

We constructed plasmids for the *IGF1* wild type (IGF1-Wild-T) and mutant type (IGF1-Mutant-C), and we inserted the +/-100bp base before and after the mutation site into the luciferase reporter vector PGL3-basic plasmid to test whether the fragment possesses initiation activity. The overexpression plasmid of transcription factor *SP1* was constructed using the pIRES2-EGFP plasmid, and we inserted the CDS region of transcription factor *SP1* into the pIRES2-EGFP plasmid. For *SP1* interference, we used Sigma’s pLKO.1-puro, which is a shRNA vector, and we designed three shRNAs and experimentally verified the three shRNA sequences shown in Supplementary Table S1. The plasmids were extracted using the QIAGEN Plasmid Midi Kit (QIAGEN, Germany).

### GC Isolation and Culture

To isolate primary granulosa cells, we extracted fresh goat ovarian follicles in a sterile Hank’s balanced salt solution. The ovarian cells were rinsed repeatedly with PBS, the cells were filtered through a 200-mesh cell sieve and collected by centrifugation, and then the primary cells were cultured in 6-cm dishes for 48 h, during which the cell culture medium was changed once to ensure cell survival and the cells were transferred to 10-cm dishes for subsequent experiments. The HEK293T cell lines were obtained from previous cultures in the laboratory, and the cell culture conditions were 37°C, 5% CO_2_, and 95% air saturation humidity. Both goat pellet cells and HEK293T cell lines were cultured using DMEM (Thermo Fisher, USA) with 10% fetal bovine serum and 100 U/ml penicillin/streptomycin. HEK293T cell lines were used to assay luciferase activity.

### Transfection and Dual-Luciferase Reporter Assay

Transfection was performed using Lip 2000 (Thermo Fisher, USA), and a dual-luciferase activity assay was performed on 24-well plates, and HEK293T cells were transfected when their density grew to about 70%. Opti-DMEM (Thermo Fisher, USA) was used for transfection, and the cells were changed 6 h after transfection and were collected and processed after 48 h of incubation to determine luciferase activity. Goat primary pellet cells were used for the gene expression assay. The cells were transfected in 6-well plates until the pellet cell density reached about 70%, and the cells were changed after 6 h. The cells were collected after 48 h of incubation for RNA extraction.

### Ethynyldeoxyuridine Incorporation Assay

Primary GCs were cultured in 96-well plates. Cell proliferation was detected after transfection with the CCK-8 Cell Proliferation and Cytotoxicity Assay Kit (Solarbio, China). After transfection treatment of the cells, the solution was changed after 6 h, and 10 µl of the CCK-8 reagent was added to each well. After incubation in the cell incubator for 2 h, the optical density values were detected at 450 nm with a microplate reader (Thermo Fisher, USA) at 0, 6, 12, 24, and 48 h. EdU was used to determine the proliferation status of granulocytes using the EdU-488 Cell Proliferation Assay Kit (Beyotime, China) according to the manufacturer’s guidelines. Using a 6-well plate, the final concentration of EdU was added to each well at 10 µM, and incubation was continued for 6 h in a cell culture incubator. The, the cells were washed three times with PBS (Thermo Fisher, USA), then fixed with a fixative for 15 min at room temperature, the fixative was removed, and the cells were rinsed three times using washing solution. 1 ml of 0.3% Triton X-100 PBS was added and incubated for 15 min at room temperature to increase cell membrane permeability of the cell membrane. In addition, 500 µl of the prepared Click reaction solution was added to each well, and the cells were incubated at room temperature for 30 min in the dark to observe and quantify the number of cells stained with EdU. Three fields were randomly selected for statistical analysis.

### Electrophoretic Mobility Shift Assay

The nuclear extracts were prepared from goat ovaries using the Nuclear Protein Extraction Kit (Beyotime, China) according to the manufacturer’s instructions. The EMSA test uses the EMSA kit (Thermo Fisher, USA), and biotin-labeled probes were synthesized by the company (Saville, China), and the biotin-labeled probe sequences are shown in Supplementary Table S2. All experimental steps were performed according to the manufacturer’s instructions.

### Statistical Analysis

PopGenev 1.31 software (http://www.ualberta.ca/∼fyeh/fyeh) was used. Allele frequencies, heterozygosity, and polymorphic information content were calculated. The method of association analysis between different genotypes and the number of lambing were analyzed using a general linear model. All results are shown as mean ± SEM. Statistical analyses were performed using IBM SPSS Statistics 25 statistical software (SPSS Inc., USA) using independent sample *t*-test.

## Results

### Association Between *IGF1* g.64943050T>C Mutation and Litter Size and Population Genetic Analysis

In our study, the *IGF1* g.64943050T>C mutation locus was present in three genotypes (TT, TC, and CC) in Yunshang black goat, with a detection rate of >90% in 400 samples, in which the dominant genotype was CC and the dominant gene was C. Population genetic statistics showed that the mutation was moderately polymorphic in Yunshang black goat (0.25<*PIC* < 0.5). The chi-square fitness test showed that the distribution of this SNP was in Hardy–Weinberg equilibrium (*p* > 0.05) (Table 2).

**TABLE 2:** Population polymorphism analysis of locus in goat.

Locus	Genotype	Genotype frequency	Allele	Allele frequency	*PIC*	*He*	*Ne*	*P*
*IGF1*g.64943050T>C	TT(43)	0.12	T	0.32	0.34	0.44	1.77	0.10
	TC(143)	0.40	C	0.68				
	CC(175)	0.48						

Note: *PIC*, *He*, and *Ne* represent polymorphism information content, heterozygosity, and effective number of alleles, respectively; *p* > 0.05 indicates the locus was under Hardy–Weinberg equilibrium.

The association analysis between g.64943050T>C and the kidding number of Yunshang black goats was performed, and the results are shown in Table 3. The kidding number of goats with the CC genotype (2.206 ± 0.044, 2.254 ± 0.056) individuals was significantly higher than that of the TC genotype (1.832 ± 0.049, 1.982 ± 0.061) and TT genotype (1.860 ± 0.090, 1.968 ± 0.117) (*p* < 0.05). In the third litter, the number of kids born of the CC genotype (2.355 ± 0.057) was significantly higher than that of the TT genotype (2.000 ± 0.107) (*p* < 0.05). The mean value of kidding number in all three litters was also significantly higher in the CC genotype (2.251 ± 0.031) than in the TC (1.921 ± 0.034) and TT (1.924 ± 0.062) genotypes. These results suggest that the *IGF1* g.64943050T>C mutation was involved in the regulation of the kidding number in Yunshang black goats.

**TABLE 3:** Least squares means and standard errors of litter size in black goats with different genotypes.

Locus	Genotype	Number	1st parity litter size	2nd parity litter size	3rd parity litter size	Average litter size
*IGF1*g.64943050T>C	TT	43	1.860 ± 0.090a	1.968 ± 0.117a	2.000 ± 0.107a	1.924 ± 0.062a
	TC	143	1.832 ± 0.049a	1.982 ± 0.061a	2.210 ± 0.063ab	1.921 ± 0.034a
	CC	175	2.206 ± 0.044b	2.254 ± 0.056b	2.355 ± 0.057b	2.251 ± 0.031b

Note: Different small letters in the same group mean significant difference (*p* < 0.05).

**FIGURE 1:**
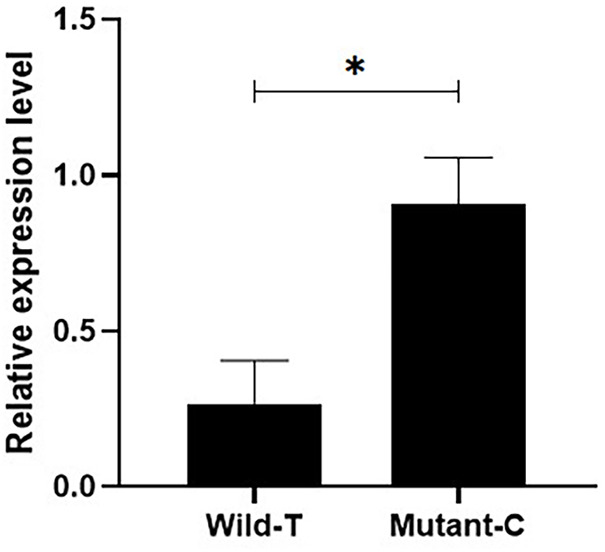
Detection of the expression of different genotypes of *IGF1* in goat ovaries. Bars represent the mean ± SEM of at least three repeats. *p < 0.05; **p < 0.01.

In addition, we quantified the expression of *IGF1* in the ovaries of Yunshang black goats from pure individuals with different genotypes, and the results showed that the *IGF1* expression was significantly higher in the individuals with the C mutation than in those with wild type T (Figure 1) (*p* < 0.05).

### Effect of g.64943050T>C Mutation on the Promoter Activity of *IGF1*

**FIGURE 2:**
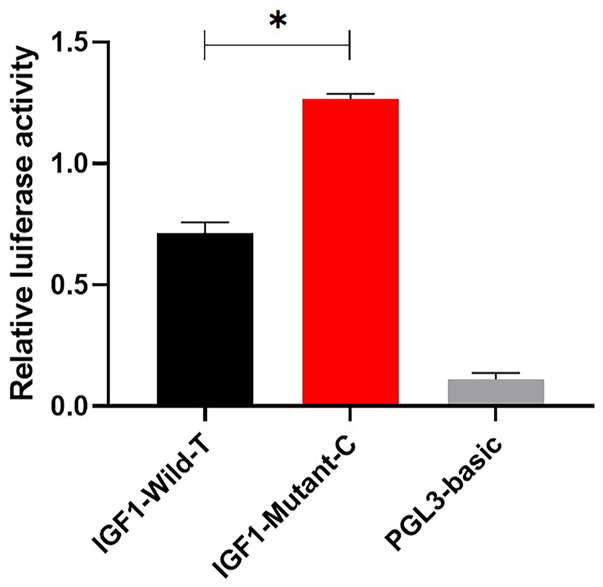
Effect of mutations on the promoter activity of *IGF1.* IGF1-Mutant-C represents the mutant type, IGF1-Wild-T represents the wild type, and PGL3-basic represents control. They were transfected into 293T cells. The results are expressed as the mean ± SEM (*n* = 3) in arbitrary units based on firefly luciferase activity normalized against Renilla luciferase activity. A t-test was conducted using SPSS 25.0 to detect the differences. Bars represent the mean ± SEM of at least three repeats. *p < 0.05; **p < 0.01.

**FIGURE 3:**
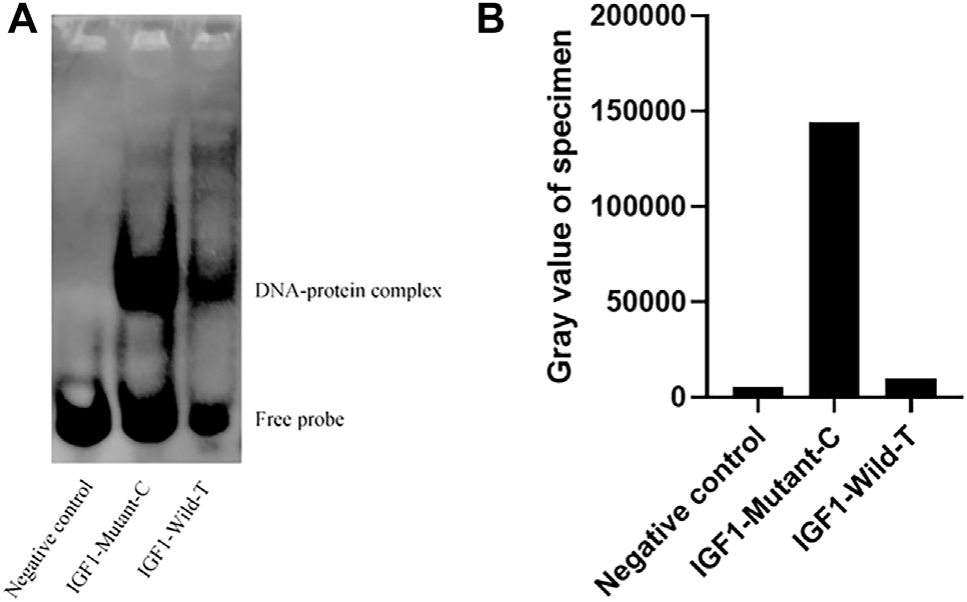
Binding of different biological probes to ovarian nucleoprotein. **(A)** Binding of the probe to the nucleoprotein. These different probes were incubated with ovary nuclear extracts, and DNA–protein complexes were visualized by autoradiography. DNA–protein complex: DNA and transcription factor SP1 binding complex. **(B)** Sample gray value.

To investigate the effect of g.64943050T>C mutation on the promoter activity of *IGF1*, the IGF1-Wild-T and IGF1-Mutant-C were constructed and transfected into HEK293T cell lines. The results showed that the luciferase activity of the IGF1-Mutant-C group was significantly higher than that in the IGF1-Wild-T group (Figure 2). The EMSA analysis showed that the bio-probe IGF1-Mutant-C significantly bound more proteins and formed more DNA–protein complexes compared to the bio-probe IGF1-Wild-T (Figure 3).

### Mutation of g.64943050T>C Adds a New Transcription Factor Binding Site

Jaspar online software was used to predict the transcription factor binding site of g.64943050T>C mutation of the *IGF1* promoter region. The result showed that transcription factor SP1 was one of the transcription factors that obtained the highest score (Supplementary Figure S2).

### Transcription Factor *SP1* Affects the Expression Abundance of *IGF1*

**FIGURE 4:**
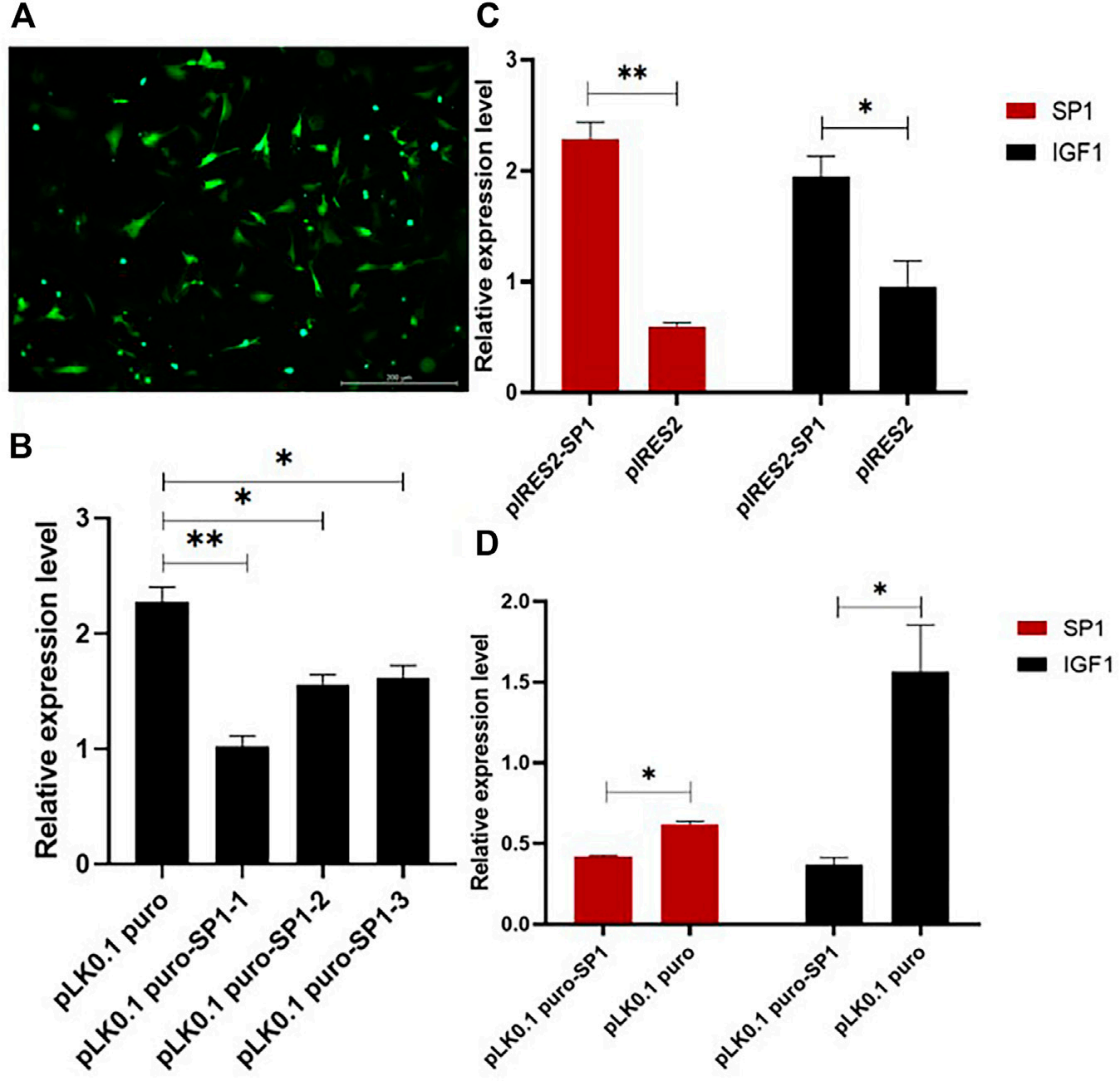
Transcription factor *SP1* affects the expression of *IGF1*. **(A)** Transfection efficiency of the vector in granular cells. **(B)** Three SP1 interference efficiency graphs were represented using RT-qPCR to detect the expression level of SP1. **(C,D)** SP1 overexpression and interference vectors were transfected into goat granulosa cells, and the expression levels of SP1 and IGF1 were detected by RT-qPCR. Bars represent the mean ± SEM of at least three repeats. *p < 0.05, **p < 0.01.

To investigate the role of transcription factor *SP1* in the transcription process of *IGF1*, the overexpression and interference vectors for transcription factor *SP1* were constructed by pIRES2-SP1 and pLK0.1 puro-SP1, respectively. One of the best interference efficiencies of the interference vector was chosen, and the transfection efficiency is shown in Figure 4A. The RT-qPCR results showed that the overexpression of *SP1* in goat GCs transfected with pIRES2-SP1 was significantly higher than that in the control group (*p* < 0.01) (Figure 4C). Correspondingly, the expression of *IGF1* was also significantly higher in goat granulosa cells transfected with pIRES2-SP1. Similarly, the expression levels of *SP1* and *IGF1* were significantly lower in goat GCs transfected with pLK0.1 puro-SP1 than those in the control group (*p* < 0.05) (Figure 4D). These results suggest that the transcription factor *SP1* is involved in regulating the expression of *IGF1* in goat GCs.

### Transcription Factor SP1 Affects the Proliferation of GCs

**FIGURE 5:**
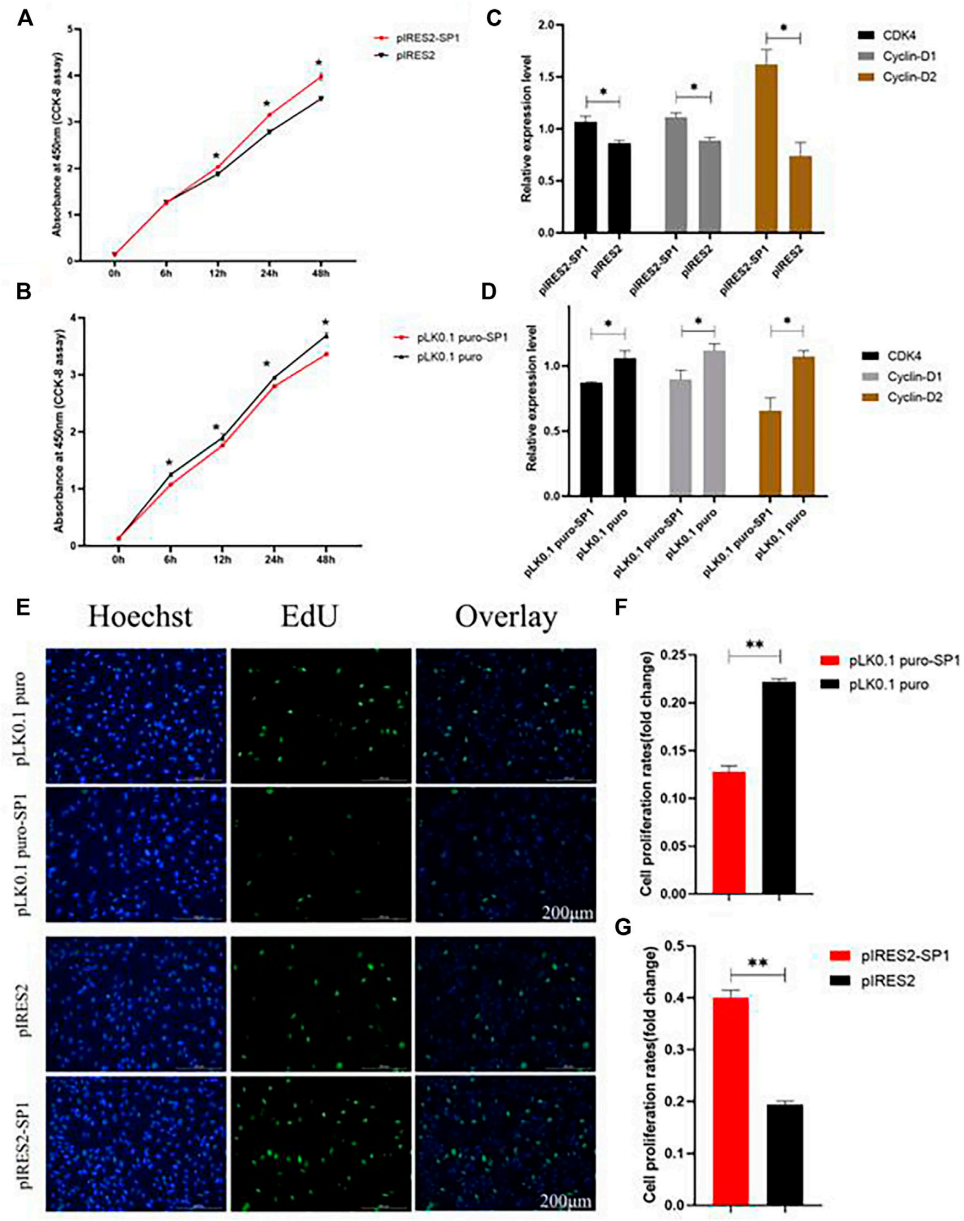
Transcription factor *SP1* regulates the proliferation of granulosa cells. **(A)** After SP1 was overexpressed, the cell counting kit-8 (CCK-8) was used to determine the proliferation curve of granulosa cells. **(B)** After interfering with SP1, Cell Counting Kit-8 (CCK-8) was used to determine the proliferation curve of granular cells. **(C)** Expression abundance of cell proliferation–related genes (*CDK4*, cyclinD1, and cyclinD2) was detected after overexpression of SP1 by RT-qPCR. **(D)** Expression abundance of cell proliferation–related genes (*CDK4*, cyclinD1, and cyclinD2) was detected after interference with SP1 by RT-qPCR. **(E)** Granulosa cells stained positive for 5-ethynyl-2-deoxyuridine (EdU) were detected with an EdU kit after SP1 overexpression and inhibition, EdU (red), Hoechst (blue). **(F,G)** Fold change of granulosa cell proliferation rate after SP1 overexpression and inhibition. Bars represent the mean ± SEM of at least three repeats. *p < 0.05; **p < 0.01.

To further investigate the effect of transcription factor SP1 on goat reproduction, we transfected pIRES2-SP1 and pLK0.1 puro-SP1 into goat ovarian GCs to detect the effect of SP1 on GC proliferation. The results of both CCK-8 and EdU showed that the proliferation rate of GCs transfected with pIRES2-SP1 was higher in GCs than in the control group (Figures 5A,E). Similarly, the proliferation rate of GCs transfected with pLK0.1 puro-SP1 was lower than that of the control group (Figures 5B,E). To further verify the effect of transcription factor *SP1* on the proliferation of goat GCs, we detected the expression of the cell proliferation factors using RT-qPCR. The results showed that the expression of *CDK4*, *Cyclin-D1*, and *Cyclin-D2* was significantly higher in GCs transfected with pIRES2-SP1 than that in the control group (Figure 5C), and similarly, the cell proliferation factors were all significantly lower in GCs transfected with pLK0.1 puro-SP1 than that in the control group (Figure 5D). All these results indicated that the transcription factor *SP1* had an effect on goat GC proliferation.

## Discussion

*IGF1*, as a member of the IGF superfamily, is an important growth factor that plays an important role in a variety of physiological processes, including reproduction, fetal development, and growth in mammals (Shen et al., 2005; Velazquez et al., 2008). Studies have shown that *IGF1* is closely associated with gestation time (Echternkamp et al., 2004), twin ovulation (Velazquez et al., 2005), preimplantation embryonic development (Patton et al., 2007), and conception rate (Yilmaz et al., 2005), while it has also been reported that polymorphisms in IGF1 are associated with reproductive traits in multiple species, such as sheep (Li et al., 2006), cattle (Andrade et al., 2008; Kim et al., 2009), chicken (Bian et al., 2008), and humans (Vella et al., 2008). In addition, *IGF1* plays an important role in the regulation of many hormones that are essential for reproduction. Mammalian reproduction is mainly controlled by gonadotropins (LH and FSH), and *IGF1* promotes gonadotropin function by enhancing the function of gonadotropin receptors (Scaramuzzi et al., 2006), while *IGF1* also stimulates ovarian function and steroidogenesis through the secretion of gonadotropin-releasing hormone (Luginbuehl et al., 2013). IGF1 is able to act directly through its receptors in the ovary. Activation of IGF1R stimulates ovarian estradiol and luteinizing hormone production (Demeestere et al., 2004).

In this study, a mutation located in the promoter region of *IGF1* was identified in goats. Previous studies have shown that polymorphisms in the 5’ regulatory region and the CDS region of the *IGF1* were associated with lambing number in four sheep breeds, namely, the Small-Tail Han sheep, the Hu sheep, Texel, and Dorset ewes, which were significantly associated with lambing number (He et al., 2012). In this study, it was found that the *IGF1*g.64943050T>C locus was significantly associated with the litter size produced in the first three litters of goats. In the first two litters, the litter size produced by CC individuals was 0.374 and 0.224 higher than that of TC and TT individuals (Table 3). In the third litter, the litter size of the CC type individuals was 0.335 higher than that of the TC type individuals (Table 3). Similarly, the average number of kids born in the first three litters was significantly higher in the CC genotype than that in the TC and TT genotypes. This suggests that *IGF1* could be a candidate molecular marker for high-fertility goat breeding. Furthermore, the expression of *IGF1* in the ovaries of homozygote individuals with different genotypes was detected, and the results showed that *IGF1* was differentially expressed between high- and low-yielding goat groups, which indicated that *IGF1* was an important candidate gene related to the goat prolificacy trait (Figure 1).

To further investigate the effect of mutations of the *IGF1* 5′ regulatory region on reproductive performance of goats, we predicted the transcription factors bound near the mutation site and found that the mutation of T>C created a new binding site for transcription factor *SP1*. Gene expression is a complex process, and transcription initiation is crucial for gene expression (de Klerk and t Hoen, 2015; Haberle and Stark, 2018). The transcription factors play an important role in eukaryotic transcription initiation. Transcription factor SP1 (specificity protein 1), a commonly expressed transcription factor, is widely expressed in mammals, and the family members include SP2, SP3, and SP4 (Suske, 1999). Transcription factor *SP1* was initially found in HeLa cell lines and is bound to multiple GGGCGG sequences (GC box) in the 21 bp repeat element of SV40 and activated *in vitro* transcription of the SV40 early promoter (Dynan and Tjian, 1983a; Dynan and Tjian, 1983b; Gidoni et al., 1984; Gidoni et al., 1985). Several studies have demonstrated that the transcription factor *SP1* is important for cell growth, differentiation, apoptosis, and oncogenesis (Kadonaga et al., 1987; Vizcaíno et al., 2015). We speculated that the *IGF1* T>C mutation leading to promoter activity change may be associated with the newly generated binding site of transcription factor *SP1*. Therefore, overexpression and interference treatment of transcription factor *SP1* were constructed into vectors and then transfected into goat GCs. The results showed that the expression of *IGF1* was significantly increased in the goat GCs with the *SP1* overexpressed group (Figure 4C), while the expression level of *IGF1* was significantly reduced in the goat GCs with the siR-SP1 group (Figure 4D). This indicated that the expression of transcription factor *SP1* significantly influenced the expression of *IGF1* in goat GCs.

The results of EdU and CCK-8 transfected with overexpressed *SP1* and siR-SP1 suggested that the transcription factor SP1 may affect the proliferation of goat GCs by influencing the expression of *IGF1* and, thus, the proliferation of GCs. It has also been previously shown that *IGF1* increased the rate of reproductive hormone production and had an effect on cell growth with specific reproductive structures (McMurtry, 1998). In mammals and birds, *IGF1* has been shown to promote mitosis in ovarian GCs, luteal GCs, and follicular membrane cells (McMurtry, 1998). In antral follicles, IGFs stimulate granulosa cell proliferation and steroidogenesis in most mammals (Silva et al., 2009). These studies further corroborate our results. *CDK4*, *Cyclin-D1*, and *Cyclin-D2* are the common cell proliferation factors (Jiang et al., 2021). In this study, the results showed that the expression of *CDK4*, *Cyclin-D1*, and *Cyclin-D2* was significantly higher in GCs transfected with pIRES2-SP1 than that in the control group (Figure 5C), and the opposite trend was observed in GCs transfected with pLK0.1 puro-SP1. Overall, the transcription factor *SP1* influenced the proliferation of goat GCs by regulating *IGF1*.

**FIGURE 6:**
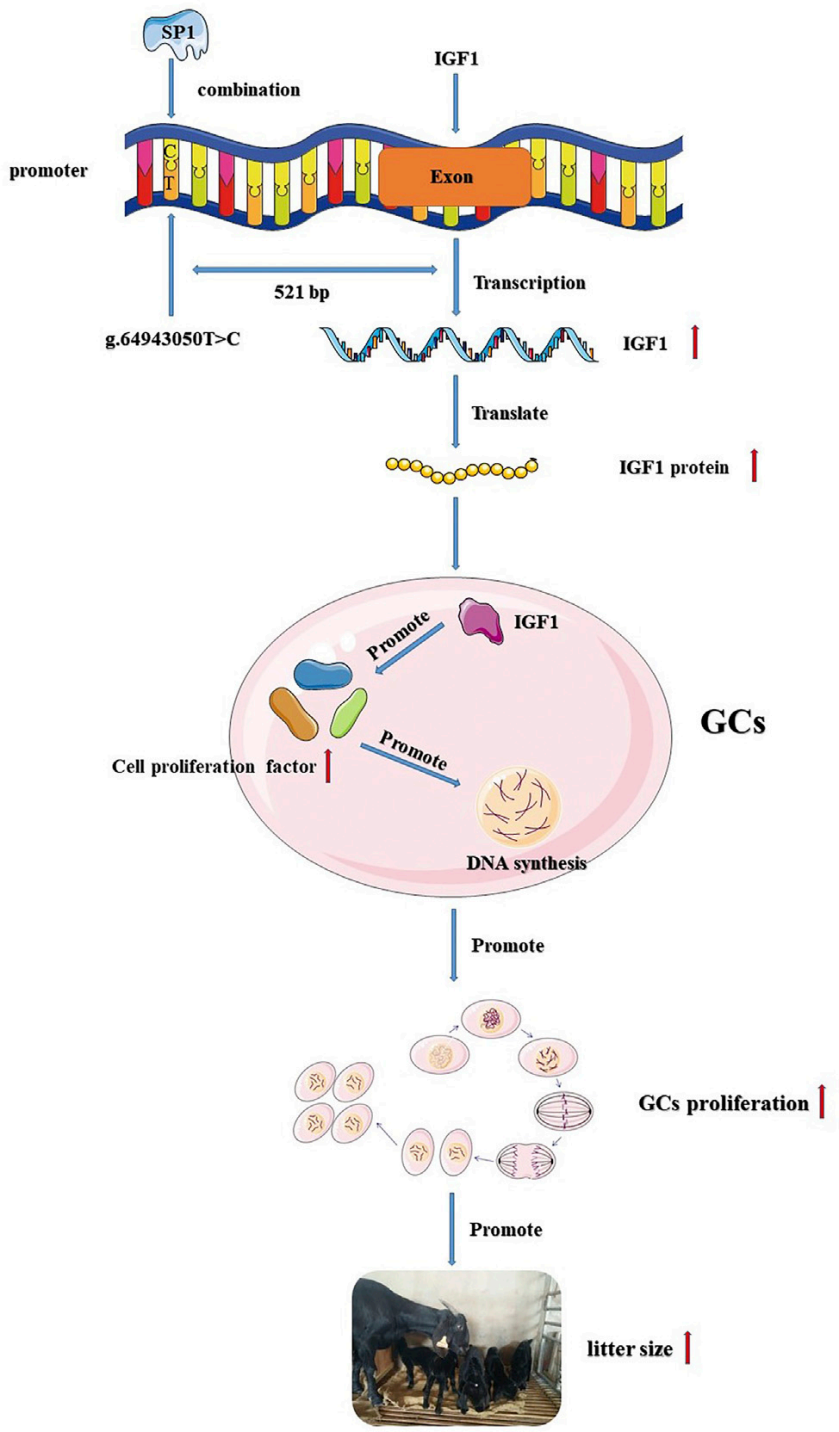
Flow chart of the *IGF1* g.64943050T>C mutation regulating goat reproduction through the transcription factor *SP1.*

In summary, we identified a T>C mutation in the promoter region of *IGF1*, which resulted in an increase in *IGF1* promoter activity. The mutation also created a new binding site for transcription factor *SP1*, and the increased activity of the *IGF1* promoter may be associated with *SP1*. Transcription factor *SP1* affected goat GC proliferation by influencing the expression of *IGF1*, and *IGF1* was associated with ovulation and the kidding number of goats (Figure 6). This study provides a new insight for goat breeding.

## Data Availability

The original contributions presented in the study are included in the article/Supplementary Material, further inquiries can be directed to the corresponding authors.

## Supplementary Material

Supplementary Figure S1 KASP typing results of *IGF1*. Blue represents the TT genotype, green represents the TC genotype, and red represents the CC genotype.

Supplementary Figure S2 Transcription factor prediction results.

Supplementary Table S1 Transcription factor *SP1* interferes with the shRNA sequence.

Supplementary Table S2 EMSA probe sequence.
