# Supplementary figures and images for: A Novel SNP in the Promoter Region of IGF1 Associated With Yunshang Black Goat Kidding Number via Promoting Transcription Activity by SP1
Source: Front Cell Dev Biol. 2022 May 12;10:873095. doi: 10.3389/fcell.2022.873095 (PMC9133608; doi:10.3389/fcell.2022.873095)

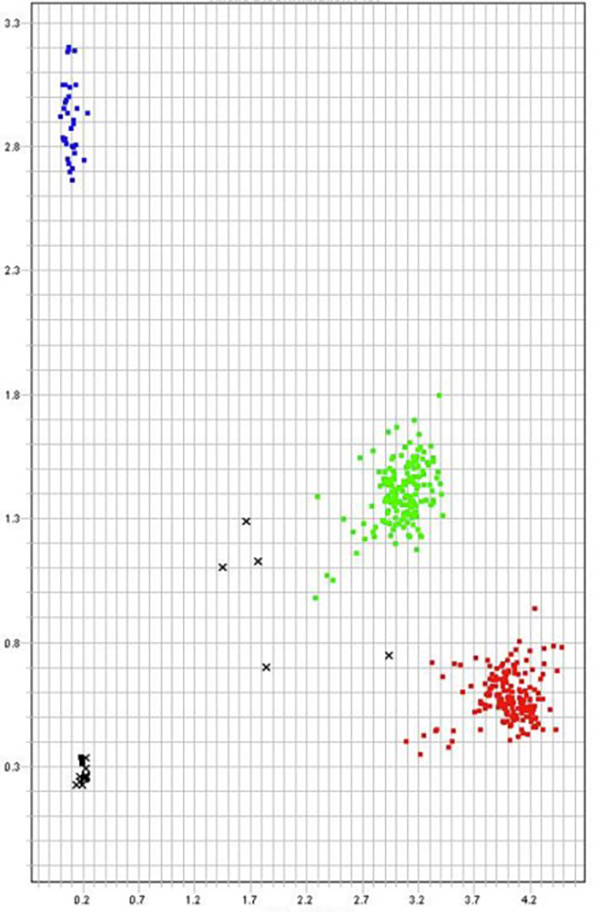

Supplement: Supplementary file 2 [file Image1.JPEG]

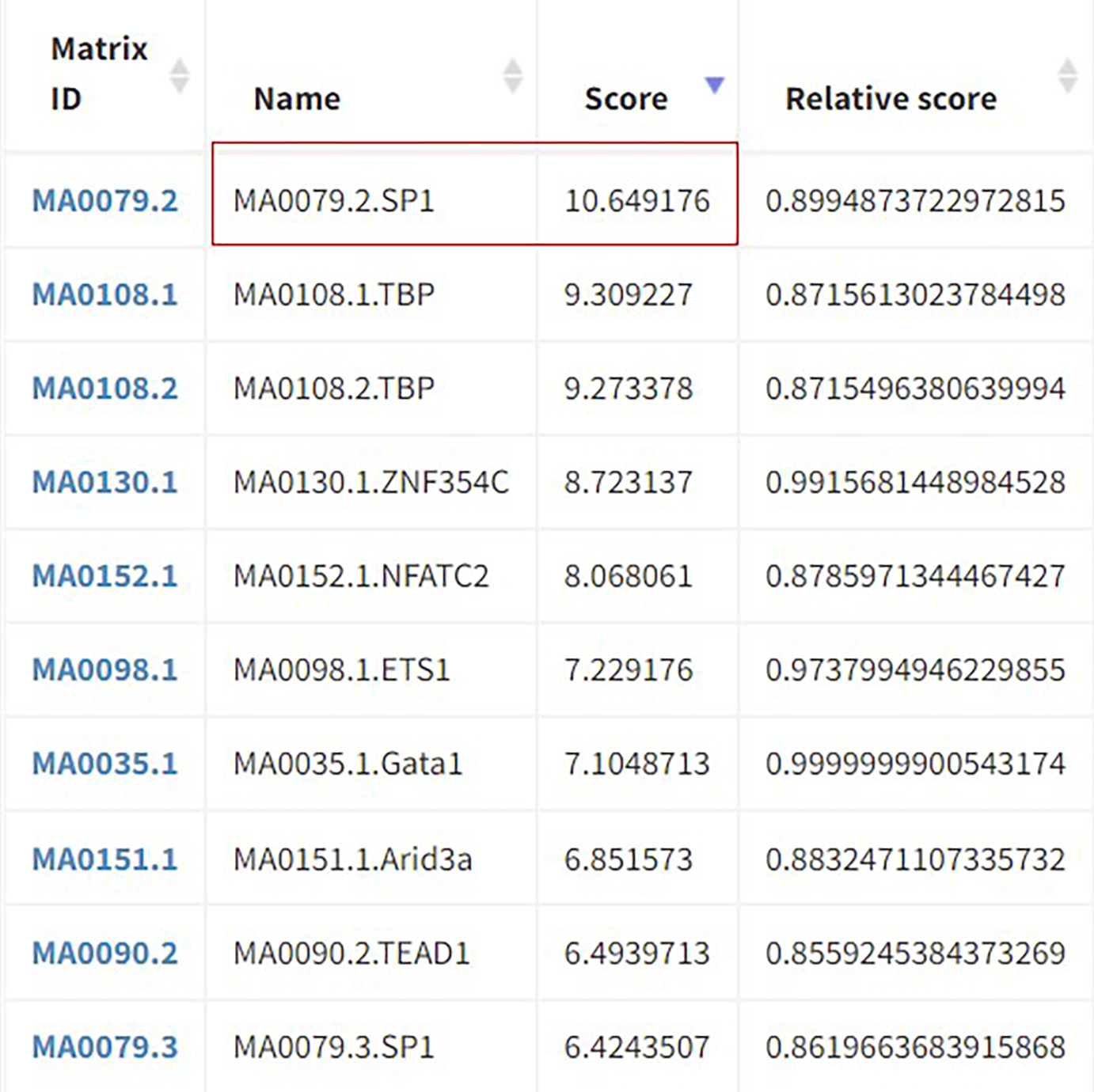

Supplement: Supplementary file 3 [file Image2.JPEG]
